# Onychomatricoma Presenting as a Dystrophic Right Great Toenail: Case Report and Review

**DOI:** 10.7759/cureus.7946

**Published:** 2020-05-03

**Authors:** Laura S Romero, Helen Park, Noushin Shoaee, Philip R Cohen

**Affiliations:** 1 Dermatology, University of California - San Diego, San Diego, USA; 2 Medicine/Dermatology, Veterans Administration Medical Center, San Diego, USA; 3 Dermatology, University of California San Diego School of Medicine, San Diego, USA; 4 Podiatry, Carmel Valley Foot and Ankle Surgery, San Diego, USA; 5 Dermatology, San Diego Family Dermatology, National City, USA

**Keywords:** hallux, matrix, nail, onychomatricoma, toenail

## Abstract

Onychomatricoma is a rare, benign nail matrix tumor. It most frequently occurs on one of the first three fingers of the dominant hand or the big toe in middle-aged women. Our patient presented with a 10-year history of a progressive thickening of her right great toenail; it bled easily and was intermittently painful. She had experienced trauma to the nail prior to the onset of the dystrophy. MRI primarily showed inflammation localized to the nail bed without bony extension. Excisional biopsy, which included both the nail plate and matrix, established the diagnosis of onychomatricoma originating from the ventral nail matrix (lunula). Nail trauma or fungal infection may have a causative role in the pathogenesis of onychomatricoma. The nail plate can show splitting, increased curvature, or ridging; it can also present with yellow, red or brown, linear, pigmented bands. The clinical differential diagnosis of onychomatricoma includes fibrokeratoma, melanonychia, onychomycosis, periungual fibroma, and squamous cell carcinoma. Dermoscopic imaging shows parallel lesion edges and splinter hemorrhages; these dermoscopic features support the diagnosis of onychomatricoma over squamous cell carcinoma. Imaging such as ultrasound or MRI may suggest the diagnosis. Biopsy of the tumor is necessary to establish the diagnosis; the tumor may derive either from the ventral nail matrix (lunula) or from the ventral surface of the proximal nail fold. Histologic features vary depending not only on tumor origin but also on tissue orientation. Proximally, there is a fibroepithelial tumor consisting of fibrous stalk pierced by epithelial invaginations; the epithelium shows matrical differentiation containing basal and prekeratogenous cells. Distally, the tumor pierces the nail plate as glove-finger digitations; these digitations appear as discrete villi in the nail plate or show their negative image as multiple empty channels that have been described as “worm holes”. The channels may be epithelial lined and contain serous fluid. It is important to obtain an adequate biopsy specimen; the distinctive fibroepithelial histology might be inapparent in partial specimens lacking the epithelial invaginations. Immunohistochemical staining can distinguish onychomatricoma from tumors that can mimic its pathologic changes: fibromyxoma, neurofibroma, and perineurioma. Complete surgical excision is generally curative.

## Introduction

Onychomatricoma is a rare, benign fibroepithelial tumor originating from the nail matrix. It was first described in 1992 by Baran and Kint [1]. To the best of our knowledge, fewer than 200 cases have been reported [2].

Patients with onychomatricoma have ranged from 4 to 72 years of age at diagnosis; however, onychomatricoma most frequently presents in middle-aged Caucasian women [3,4]. Although the etiology of onychomaticoma remains to be determined, the tumor has been associated with trauma and onychomycosis [5]. The fingernails of the dominant hand are most frequently affected, particularly the thumb, and second and third fingers, while the nail of the great toe is the most commonly involved toenail [3-6].

The features of onychomatricoma presenting on the right great toe of a middle-aged woman are described. Her tumor arose from the ventral nail matrix (lunula), and it persisted after an incomplete excision. The characteristics of this nail tumor are also reviewed.

## Case presentation

A 43-year-old woman presented with a 10-year history of throbbing pain and progressive thickening of the right great toe. She also experienced easy bleeding of the toe when she would file the nail. The patient had traumatized the toe prior to the onset of symptoms.

**Figure 1:**
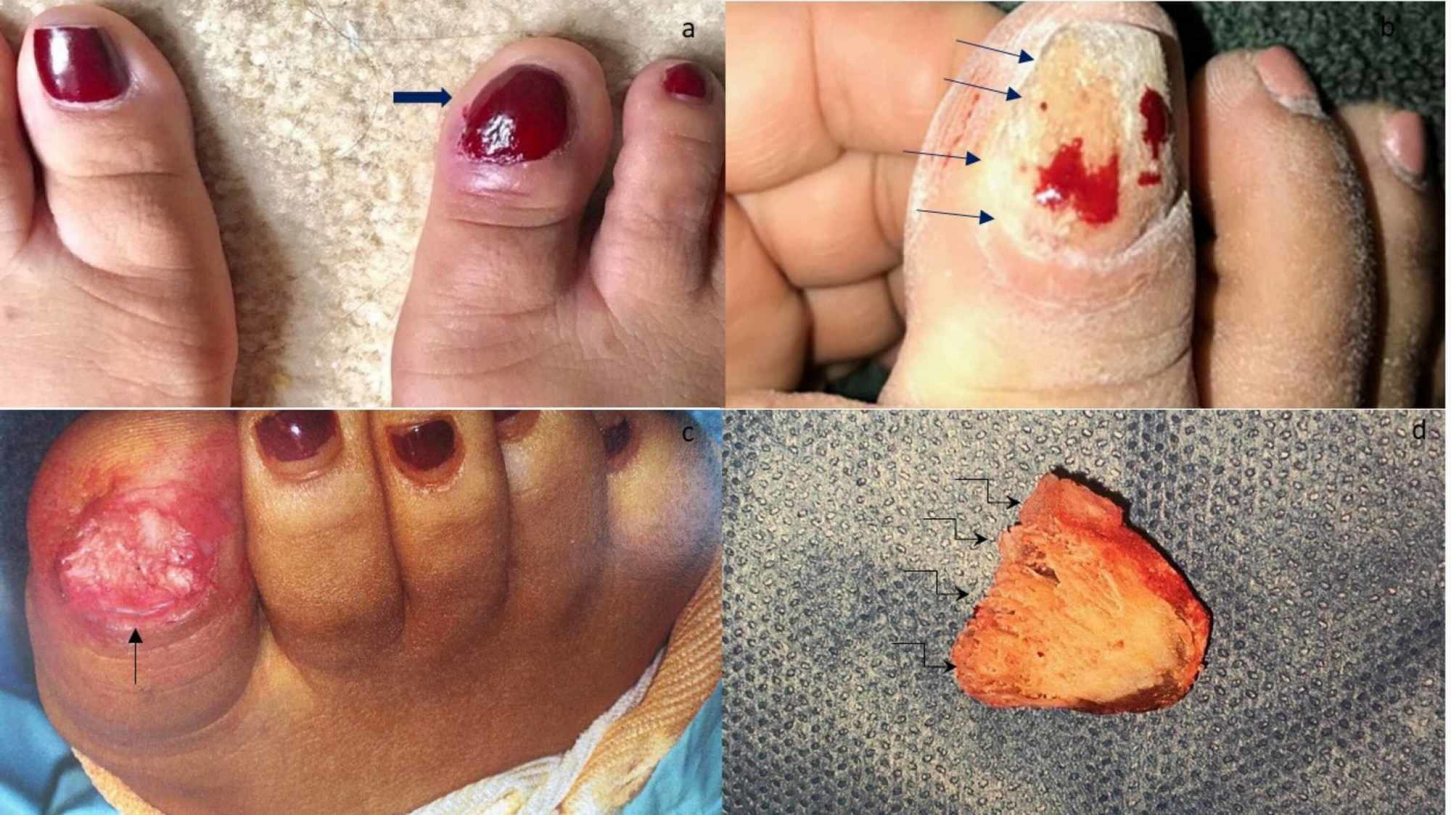
Onychomatricoma of the right great toenail of a 43-year-old woman There is nail polish on both great toenails; however, the affected right great toenail (black arrow) is thickened and shows overcurvature (a). After partial removal of the nail polish, the right great toenail shows dystrophy and pitting (black arrows) (b). After the nail plate has been removed, the right great toe reveals a tumor (black arrow) with finger-like projections (c). The undersurface of the removed nail plate has numerous spicules (bent black arrows) (d).

Cutaneous examination revealed a nontender right great toe. There was diffuse nail dystrophy with proximal nail fold thickening and scale. All of her fingernails and other toenails were normal in appearance (Figure 1).

A shave biopsy was performed. Microscopic examination revealed hyperkeratosis and underlying fibrosis suggestive of scar. The specimen was negative for hyphal elements by periodic acid-Schiff (PAS) stain. 

A MRI of the toe was done to evaluate the digit. It showed moderate, focal swelling adjacent to the collateral ligament of the first metatarsophalangeal joint and mild inflammatory signal intensity of the nailbed of the first toe. No effusions were noted nor were any abnormalities to bone, joint, or cartilage.

The patient underwent an excisional biopsy three months later including both nail bed and the nail matrix down to bone. The nail plate was avulsed. A tumor with finger-like projections arising from the nail matrix was revealed (Figure 1). In addition, the nail plate had spicules.

**Figure 2:**
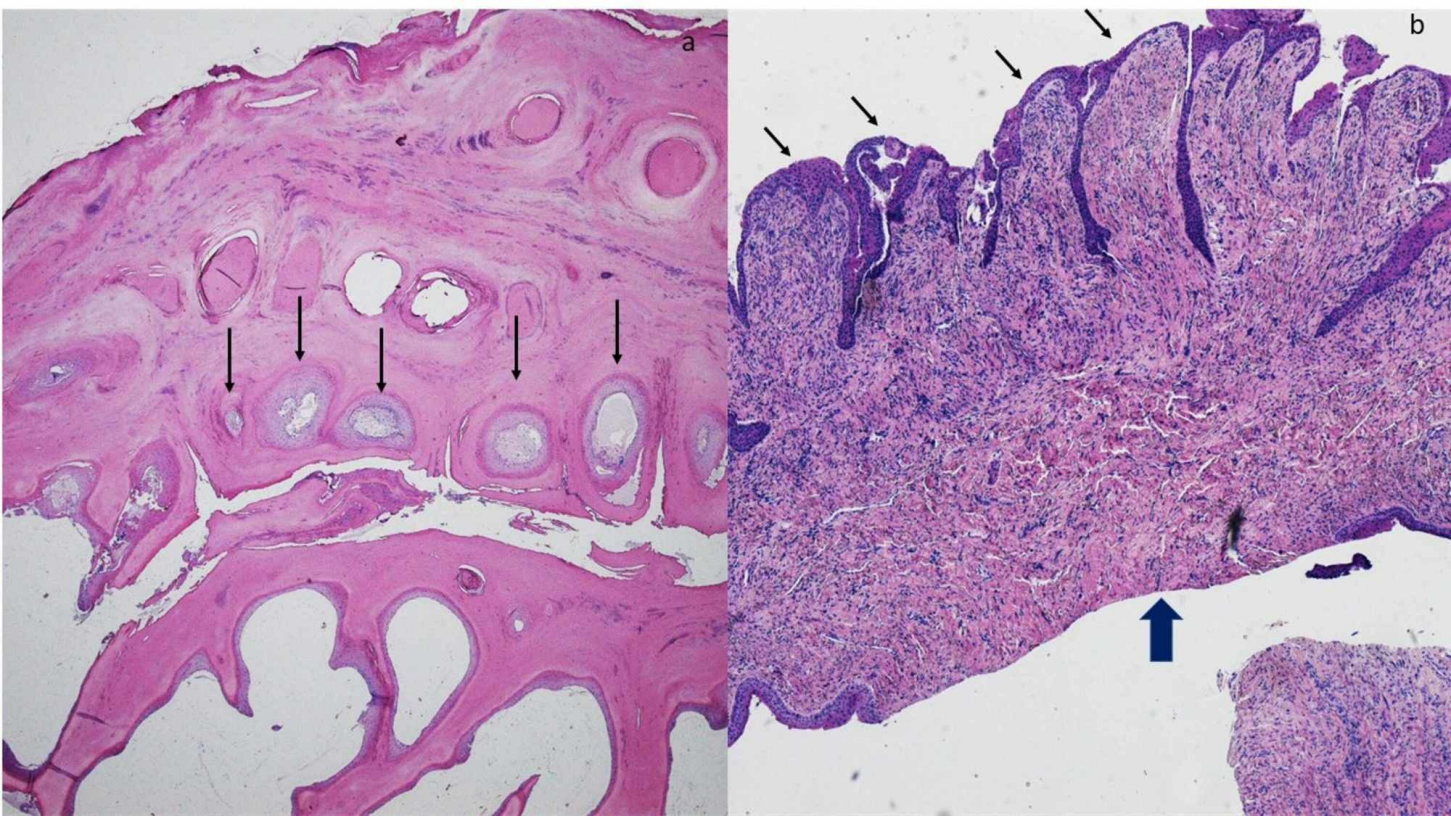
Pathology of onychomatricoma: transverse sections of distal and proximal tissue specimen The transverse sections of the distal tissue specimen (a) show several woodworm-like holes (black arrows); these are empty or serum-filled cavities lined by parakeratotic epithelium. The transverse sections of the proximal tissue specimen (b) show a pedunculated tumor; this tumor displays not only surface mamillations (thin black arrows) but also underlying fibrous stroma (thick black arrow) (hematoxylin and eosin: a, x2; b, x4).

Microscopic examination of transverse sections of the distal tissue specimen showed wormhole-like cavities lined by parakeratotic epithelium. Transverse sections of the proximal tissue specimen showed a mamillated fibrous tumor with deep epithelial invaginations (Figure 2).

**Figure 3:**
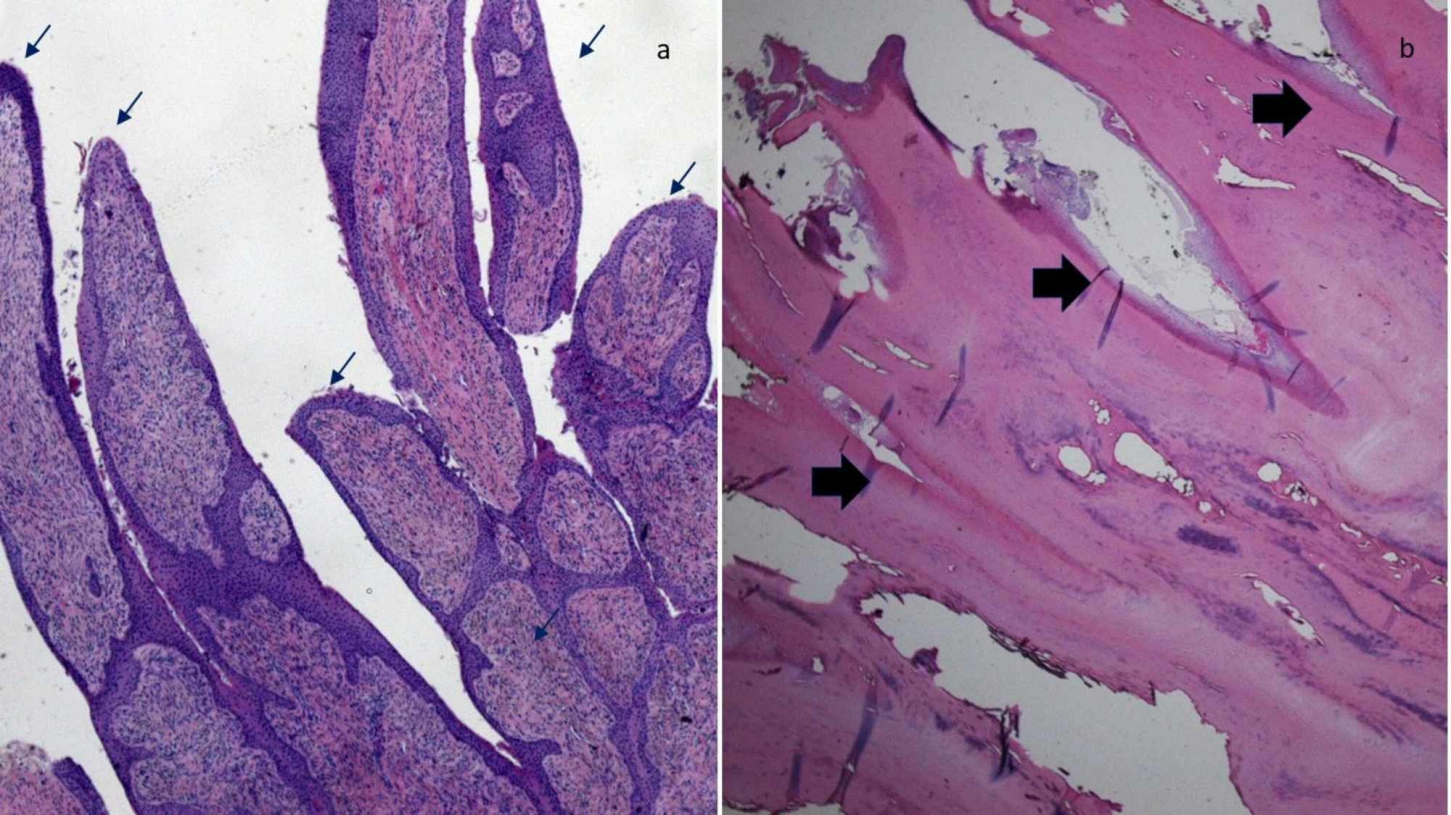
Pathology of onychomatricoma: longitudinal sections of proximal and distal sections The longitudinal sections of the proximal tissue specimen (a) show a warty tumor; the surface reveals verrucous papillations (black arrows). The longitudinal sections of the proximal tissue specimen (b) show spike-like invaginations (black arrows); the invaginations are lined by parakeratotic (matrical) epithelium (hematoxylin and eosin: a, x2; b, x4).

Microscopic examination of longitudinal sections of the proximal tissue specimen showed a pedunculated fibrous tumor coated with spike-like, epithelial-lined digitations. Longitudinal sections of the distal tissue specimen showed a nail plate with deep invaginations with retained matrical epithelium (Figure 3).

The pathologic features observed were diagnostic of the ventral nail matrix (lunula) variant of onychomatricoma. The PAS stain was negative for hyphal elements. Specimen fragmentation prevented an adequate evaluation of the margins of the excisional biopsy.

The surgical site healed without complication. Five months later, the patient returned complaining of a plaque in the right great toenail bed in the area of the prior excision. Repeat biopsy demonstrated residual onychomatricoma. She did not return for additional treatment.

## Discussion

Onychomatricoma most commonly presents with leukonychia, splinter hemorrhages, and nail plate thickening. Other nail findings may include transverse overcurvature, yellow, brown, or red bands, nail plate fractures, and prominent ridging. The clinical differential diagnosis includes fibrokeratoma, melanonychia, onychomycosis, periungual fibroma, and squamous cell carcinoma [6].

Dermoscopy can be very useful in the evaluation of a suspected onychomatricoma. Dermoscopic features that are frequently observed in onychomatricoma include longitudinal parallel white lines, parallel lesion edges, splinter hemorrhages, free-edge nail pitting, free-edge dark dots, and free-edge nail thickening. Indeed, the presence of parallel lesion edges and splinter hemorrhages may be helpful to differentiate onychomatricoma from squamous cell carcinoma which does not have these findings [6].

Ultrasound or MRI may be helpful in evaluating patients in whom the diagnosis of onychomatricoma is being considered. They show a soft tissue tumor above bone, expressed as a hypoechogenic signal on ultrasound and a low-intensity signal on MRI in the proximal nail matrix reflecting the fibrous stalk. In addition, the finger-like projections of the tumor show a hyperechogenic signal on ultrasound and a high-intensity signal on MRI [7]. These key findings may be missed by a radiologist unfamiliar with the diagnosis of onychomatricoma; our patient’s MRI report simply described increased signal intensity in the nail bed of the right big toe.

Histologic characteristics of onychomatricoma vary depending on whether the tumor is predominantly derived from the ventral nail matrix (lunula) or the ventral surface of the proximal nail fold and/or short apical matrix. The ventral nail matrix (lunula) origin is most common, and longitudinal sections show a single, gloved, spindly finger appearance comprised of a pedunculated tumor lined by matrical epithelium. Proximally, transverse sections show a single, large connective tissue tumor with a mamillated surface pierced by regular epithelial invaginations comprised of matrical epithelium containing basal and prekeratogenous cells. Distally, transverse sections show a nail plate that is markedly thickened with multiple, empty cavities, which are the negative images of the tumor’s fibrous digitations. Similar to our patient, these cavities may have retained parakeratotic epithelium and be filled with serous fluid [3,8].

The tumor’s connective tissue stroma, particularly noted on longitudinal sections, is organized into two layers. The superficial layer is loose and wavy and contains mast cells, while the deeper layer is eosinophilic, hypocellular and densely fibrocollagenous. Rarely, pleomorphic and hyperchromatic, uninucleated and multinucleated giant cells are also present [8,9].

The pathologic changes observed when the onychomatricoma is located in the ventral surface of the proximal nail fold and/or short apical matrix are different than those noted when the tumor originates from the ventral nail matrix (lunula). Longitudinal sections show a spiky, fibroepithelial tumor with deep, V-shaped invaginations. The surface epithelium shows a thick keratogenous zone at the base of each invagination. Proximally, transverse sections show an elongated fibrous tumor that follows the shape of the proximal nail fold; distally, transverse sections show numerous fibroepithelial digitations that appear as discrete villi. Similar to ventral nail matrix (lunula)-derived onychomatricoma, the nail plate in proximal nail fold-derived onychomatricoma is thickened; however, the characteristic cavities present with ventral nail matrix (lunula)-derived onychomatricoma are absent [3,8].

Establishing the correct diagnosis can be difficult if the nail plate is not also submitted with the underlying tumor. The histologic differential diagnosis, particularly for ventral nail matrix (lunula) origin onychomatricoma, includes tumors similar to onychomatricoma characterized by collagenous or myxocollagenous stroma with a proliferation of spindled fibroblasts and delicate microvasculature. These tumors include superficial acral fibromyxoma (including cellular digital fibroma), neurofibroma, and perineurioma. 

Immunoperoxidase staining may be helpful in establishing the diagnosis of onychomatricoma. Onychomatricoma stains with cluster of differentiation 34 (CD34) and does not stain with cluster of differentiation 99 (CD99), epithelial membrane antigen (EMA), and soluble protein in saturated ammonium sulfate at neutral pH 100 (S100). In contrast, superficial acral fibromyxoma stains with CD99, perineurioma stains with EMA, and neurofibroma stains with S100. However, none of these tumors contain the epithelial-lined, spiky digitations of onychomatricoma that are best visualized on hematoxylin and eosin-stained sections [8,9].

Acral fibrokeratoma may mimic proximal nail fold-derived onychomatricoma; however, acral fibrokeratoma is seldom located under the nail plate and it lacks the filamentous strands of onychomatricoma [1]. Pleomorphic fibroma, similar to onychomatricoma, may show pleomorphic and hyperchromatic, and uninucleated and multinucleated giant cells; however, pleomorphic fibroma lacks two of onychomatricoma’s characteristic features: epithelial invaginations and two distinct (wavy superficially and collagenous deep) fibrous layers [8,9]. Another nail matrix tumor, onychocytic matricoma, can clinically present similarly to onychomatricoma as longitudinal nail plate thickening; however, onychocytic matricoma shows endokeratinization, lacks a stromal component, and lacks the undulating, keratogenous zone-like epithelium characteristic of an onychomatricoma [3].

Surgical excision remains the treatment of choice for onychomatricoma. Once the tumor has been exposed, after avulsion of the nail plate, it must be completely removed. Recurrence is uncommon, yet recurrent onychomatricoma has previously been observed [9].

## Conclusions

Onychomatricoma is an uncommon nonmalignant nail matrix tumor that presents with a nail plate, which demonstrates splitting, overcurvature, and colored linear bands. The patient may have a history of fungal infection or trauma to the affected nail. The presence of splinter hemorrhages and parallel lesion edges are dermoscopic features present in onychomatricoma and lacking in squamous cell carcinoma, a malignant nail bed tumor that can morphologically mimic onychomatricoma and should therefore be excluded. Evaluation with ultrasound and/or MRI may be helpful when evaluating the patient; however, a biopsy will confirm the diagnosis. Pathologic changes on hematoxylin and eosin-stained sections of the tissue biopsy specimen and the results of immunohistochemistry studies can establish the diagnosis and exclude other subungual neoplasms.
